# Distribution characteristics of DNA vaccine encoded with glycoprotein C from Anatid herpesvirus 1 with chitosan and liposome as deliver carrier in ducks

**DOI:** 10.1186/1743-422X-10-89

**Published:** 2013-03-16

**Authors:** Kunfeng Sun, Xin Li, Jinfeng Jiang, Anchun Cheng, Mingshu Wang, Dekang Zhu, Renyong Jia, Shun Chen, Yi Zhou, Xiaoyue Chen, Xiaoyu Wang

**Affiliations:** 1 Institute of Preventive Veterinary Medicine, Sichuan Agricultural University, Wenjiang, Chengdu City, Sichuan, 611130, China; 2 Avian Disease Research Center, College of Veterinary Medicine of Sichuan Agricultural University, 46 Xinkang Road, Ya’an, Sichuan, 625014, China; 3 Key Laboratory of Animal Disease and Human Health of Sichuan Province, Sichuan Agricultural University, Wenjiang, Chengdu City, Sichuan, 611130, China

**Keywords:** DNA vaccine, Tissue distribution, Deliver carrier, Chitosan, Liposome, Anatid herpesvirus 1, Glycoprotein C

## Abstract

**Background:**

A eukaryotic expression plasmid encoding glycoprotein C (gC) of Anatid herpesvirus 1 (AnHV-1) (pcDNA3.1-gC) was constructed and validated. The tissue distribution of chitosan/DNA complexes, liposome/DNA complexes and pcDNA3.1-gC alone were evaluated using a quantitative real-time PCR based TaqMan™ probe following intramuscular administration in ducklings.

**Results:**

Compared with pcDNA3.1-gC alone, liposomes universally increased the plasmid DNA copy number at the injection sites, liver, spleen, heart, brain, bursa of Fabricius, and especially in the enteron (esophagus, duodenum, rectum, and cecum). Chitosan also universally increased the plasmid DNA copy number at the injection sites, liver, spleen, heart, brain and esophagus. Compared with lipoplex-gC, higher chitosan-gC plasmid DNA copy numbers were detected at the injection sites, liver, spleen, heart, brain and esophagus. In contrast, compared with lipoplex-gC, lower copy numbers of chitosan-gC plasmid DNA were detected in the duodenum, rectum and cecum.

**Conclusions:**

The results of this study demonstrated that chitosan and liposomes mediated rapid and extensive plasmid distribution in duck tissues, with low levels maintained from 1 d after DNA vaccination.

## Background

Anatid herpesvirus 1 (AnHV-1), belonging to the family *Herpesviridae*, subfamily *Alphaherpesvirinae*, genus *Mardivirus*, is the cause of severe epidemics of duck virus enteritis (DVE), which account for significant economic losses. DVE, also referred to as duck plague, is a highly contagious acute disease of ducks, geese, and swans, which is characterized by hemorrhagic lesions in the blood vessels, gastrointestinal mucosa, and lymphoid tissues [1-3].

As a multifunctional glycoprotein of *Alphaherpesvirinae*, glycoprotein C (gC) is involved in viral attachment, stability, virulence and other functions [4-15]. Good immune responses and protective efficacy against herpesviruses have been reported in mice and related animals following immunization with DNA vaccines based on the gC gene [16-19]. Our previous study also demonstrated that the AnHV-1 gC protein was highly immunogenic [20].

DNA vaccines, which are a new generation in the vaccine family, are defined by the expression of the exogenous genes *in vivo* through a delivery system, such as naked plasmid DNA (pDNA) [21]. However, transgene expression occurs only in transduced cells. Therefore, the tissue distribution of genes is an important factor determining the efficacy of in vivo gene transfer.

Generally speaking, the tissue distribution characteristic of an externally administered compound is determined by its interaction with the recipient, and such interaction is regulated by the physicochemical and biological properties of the compound and the anatomical and physiological properties of the host. To improve the delivery and cellular uptake of plasmid DNA after in vivo administration, a number of synthetic pDNA delivery vehicles have been developed. These vehicles can currently be divided into cationic polymers and liposomal formulations. Cationic polymers include DNA adsorbed to or entrapped within biodegradable materials such as poly-lactide-co-glycolide or chitosan. Both cationic polymers and liposomal formulations make use of the electrostatic interaction between negatively charged nucleic acids and the positive charges of the synthetic vector [22].

In our research, we developed a novel delivery system, designated pcDNA3.1-gC. A clear understanding of the tissue distribution of pcDNA3.1-gC and its complexes formed with synthetic carrier systems is a prerequisite for a strategy aimed at developing effective in vivo gene transfer methods. Here, we discuss the tissue distribution characteristics of pcDNA3.1-gC, which was systemically administered in the free and complexed forms.

## Results

### Construction and identification of pcDNA3.1-gC

**Figure 1:**
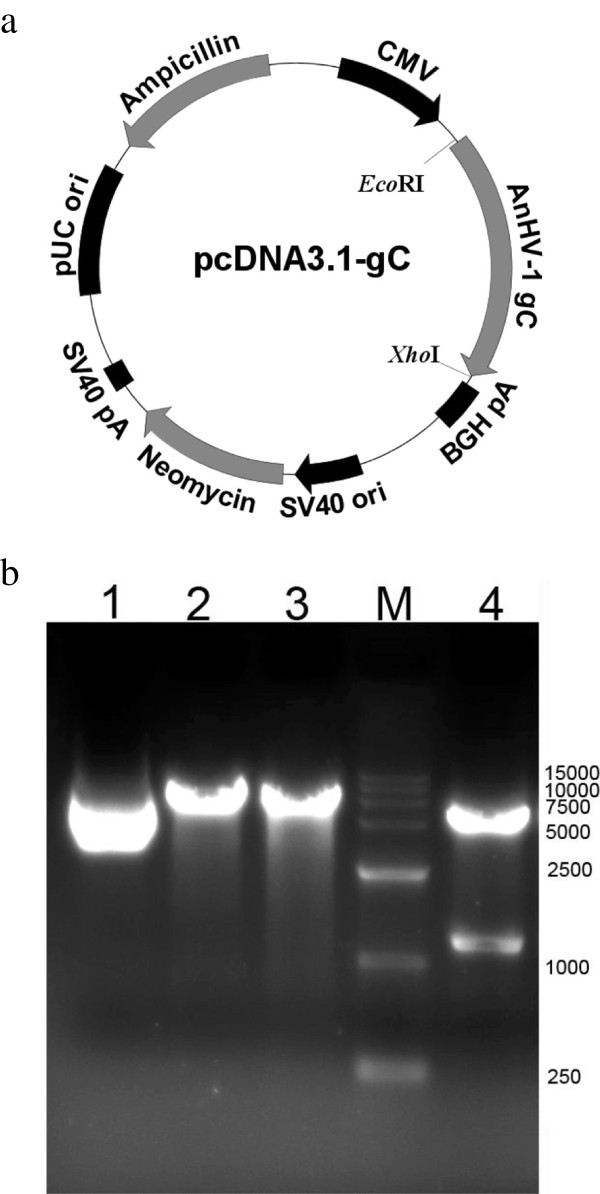
**Identification of the recombinant plasmid pcDNA3.1-gC a**) Schematic representation of pcDNA3.1-gC. The gC gene was inserted into pcDNA3.1 utilizing *Eco*R I and *Xho* I sites. **b**) The digestion products were separated by electrophoresis. Lane 1, pcDNA3.1-gC; Lane 2, single fragment after restriction enzyme digestion with *Eco*R I; Lane 3, single fragment after restriction enzyme digestion with *Xho* I; Lane M, DNA marker-DL15000; Lane 4 two fragments after restriction enzyme digestion with *Xho* I and *Eco*R I.

As shown in Figure 1a, AnHV-1 gC gene was cloned into the eukaryotic expression vector pcDNA3.1(+), resulting in the DNA vaccine plasmid pcDNA3.1-gC. DNA sequencing showed that pcDNA3.1-gC was correct. The constructed plasmid was subjected to digestion with *Eco*R I and *Xho* I. Electrophoretic separation of the digestion products (Figure 1b) showed that the construction of the recombinant plasmid was successful.

### Expression of pcDNA3.1-gC in eukaryotic cells

**Figure 2:**
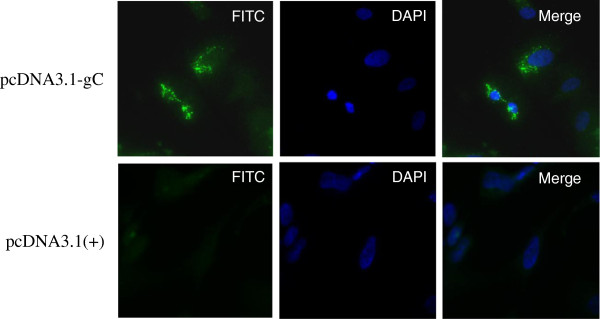
Distribution of pcDNA3.1-gC in COS-7 cells by indirect immunofluorescence assay.

As shown in Figure 2, intensive fluorescence was found in the cytoplasm of COS-7 cells transfected pcDNA3.1-gC following staining with polyclonal anti-gC hyperimmune serum. In contrast, no fluorescence was detected in COS-7 cells transfected with pcDNA3.1(+).

### Biodistribution of pcDNA3.1-gC alone

**Figure 3:**
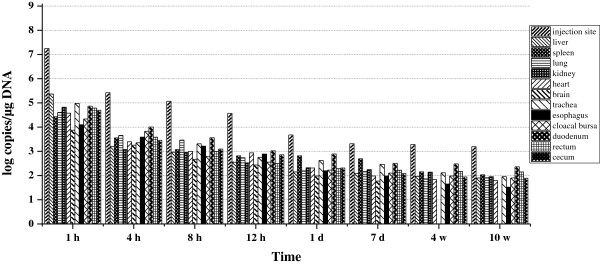
**Biodistribution of pcDNA3.1-gC in ducklings immunized with pcDNA3.1-gC alone.** Each time point represents the mean plasmid copies per microgram DNA obtained from two ducklings.

The biodistribution of pcDNA3.1-gC in duckling tissues was determined following intramuscular injection. Copy numbers were quantified in different organs at various times after intramuscular injection. As shown in Figure 3, differences in plasmid distribution were observed between organs. One hour post-injection, plasmid DNA was widely distributed among all the analyzed organs. The concentration of plasmid DNA was determined in order of the injection site (liver, kidney, lung, heart, cecum and spleen) at 1 h post-injection. However, at the highest levels, the concentrations of plasmid DNA in other tissues were 1–2 orders of magnitude lower than at the injection site. Plasmid DNA was detected in all analyzed organs from 1 h to 10 weeks post-injection. At the injection site, there was a 3.7 × 10^3^-fold reduction in the plasmid copy numbers at 1 d compared with 1 h post-injection. In other organs, there was also a significant decrease at 1 d compared with 1 h post-injection. Subsequently, the number of plasmid copies was maintained at a low level. However, higher plasmid copy numbers (approximately 10-fold) were detected at the injection site than in other organs at different times. Minimum copy numbers were found in the brain, indicating a low capacity of the plasmid to pass the blood-brain barrier.

### Biodistribution of lipoplex-gC

**Figure 4:**
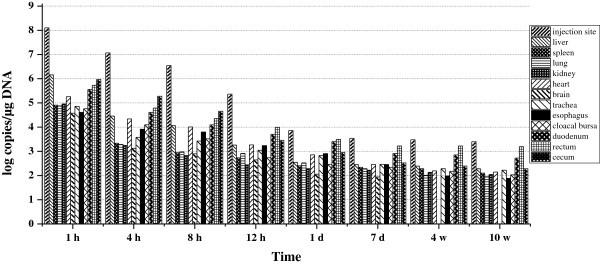
**Biodistribution of pcDNA3.1-gC in ducklings immunized with lipoplex-gC.** Each time point represents the mean plasmid copies per microgram DNA obtained from two ducklings.

As shown in Figure 4, plasmid DNA was detected in all analyzed organs from 1 h to 10 weeks post-inoculation. One hour after intramuscular administration, plasmid DNA was distributed widely in all analyzed organs. The highest plasmid DNA concentration was detected at the injection site, cecum, and liver at 1 h. Similar to pcDNA3.1-gC alone, at the highest levels, the concentrations of plasmid DNA in other tissues were still 2–3 orders of magnitude lower than those at the injection site, and the number of plasmid copies was maintained at a low level after 1 d, with minimum copy numbers detected in the brain. However, compared with pcDNA3.1-gC alone, lipids universally increased the quantity of plasmid in different organs, especially in the enteron (esophagus, duodenum, rectum, and cecum).

### Biodistribution of chitosan-gC

**Figure 5:**
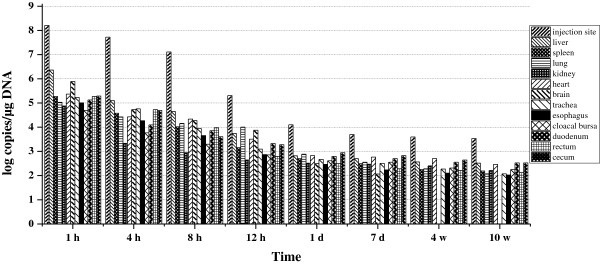
**Biodistribution of pcDNA3.1-gC in ducklings immunized with chitosan-gC.** Each time point represents the mean plasmid copies per microgram DNA obtained from two ducklings.

As shown in Figure 5, similar to pcDNA3.1-gC alone and lipoplex, chitosan-gC complexes also showed the highest organ distribution at the administration site. One hour after intramuscular administration, plasmid DNA was widely distributed in all analyzed organs. The plasmid copy numbers detected at 1 d were substantially lower than those detected at 1 h post-inoculation. In parenchymatous organs, the chitosan/DNA complex group exhibited higher plasmid DNA levels than those detected in the other groups. A notable difference in plasmid DNA levels was detected in the brain, with 39-fold higher levels in the chitosan/DNA group compared with the other groups at 1 h post-inoculation.

## Discussion

DNA immunization against AnHV-1 has been developed in our laboratory [20]. To increase the efficacy of the AnHV-1 gC DNA vaccine, DNA-lipid complexes and chitosan-DNA nanoparticles were prepared. In the present study, the fates of these complexes and pcDNA3.1-gC alone following intramuscular injection were investigated in ducklings. To our knowledge, this study is the first assessment of the organ distribution and retention time of plasmid DNA administered in lipid complexes or chitosan nanoparticles in ducklings.

More than three decades ago, Wolff showed that the injection of mouse muscle with a DNA plasmid resulted in significant expression of the protein encoded by the plasmid [21]. Progress in the field of DNA vaccination has resulted in the development and the marketing of three veterinary DNA vaccines [23]. The uptake of DNA plasmid by cells upon injection is very inefficient: only a small proportion of the injected material is internalized by cells and results in successful transfection [24]. Therefore, a number of strategies have been used for increasing DNA vaccine potency. Chemical adjuvants for enhancing plasmid DNA expression include liposomes and cationic polymers, both of which have shown promise for enhancing the expression and immunogenicity of plasmid DNA vaccines in animal models [25].

Our results showed that liposomes universally increased the plasmid DNA copy numbers at the injection site, liver, spleen, heart, brain, bursa of Fabricius, and especially in the enteron (esophagus, duodenum, rectum, and cecum), compared with pcDNA3.1-gC alone. Similarly, chitosan also universally increased the plasmid DNA copy numbers at the injection site, liver, spleen, heart, brain and esophagus. These results indicated that liposomes and chitosan enhance the distribution in tissues. In addition, compared with lipoplex-gC, chitosan-gC mediated production of higher numbers of plasmid DNA copies at the injection site, liver, spleen, heart, brain and esophagus. Nevertheless, compared to lipolex-gC, lower copy numbers were detected in duodenum, rectum, and cecum.

In terms of the effects of time after inoculation, the highest concentrations of DNA were detected all the tissues at 1 h after injection. The number of plasmid copies had decreased by 3–4 orders of magnitude at 1 day after injection. Subsequently, the number of plasmid copies was maintained at a low level and plasmid DNA was still be detected 10 weeks post-injection. Our study is consistent with other studies, which have shown that plasmid remained for several weeks following clearance of the majority of the plasmid within the first 24 h post-inoculation [26-28]. Additionally, results based on indirect immunohistochemical staining (IHC) have shown that the gC proteins are still found in the liver, bursa of Fabricius, duodenum, caecum and rectum in the intramuscular injection group at 10 weeks post-inoculation [29].

Transfection of cells by chitosan nanoparticles and lipoplexes occurs in three phases: (1) cellular uptake by formation of endosomes into the target cells; (2) release from the endosome; (3) entry of plasmid DNA into the cell nucleus [30,31]. Liposomes can protect the nucleic acid from extracellular degradation, ensuring appropriate tissue targeting, and facilitating the delivery of functional DNA into the cell [32,33]. As a cationic polymers, chitosan (CS) is an effective, naturally-occurring material used for synthesizing nanoparticles with advantageous properties such as low toxicity, low immunogenicity and excellent biocompatibility [34,35]. Accordingly, following inoculation with chitosan/DNA or liposome/DNA complexes, plasmid DNA diffuses from the injection site and/or degrades more slowly because of a liposomes or chitosan depot effect. The liposome/DNA or chitosan/DNA depot is thought to provide some protection against nucleases, thus extending the half-life of the plasmid DNA at the injection site [30]. It has been shown that formulation of DNA vaccines into liposomes enhances cellular and humoral immunity [34,35]. Our previous study also showed that chitosan significantly improved CD4^+^ and CD8^+^ T cell responses to at least 6 weeks post-injection in Balb/c mice [36]. The immune responses to these formulations and protection against Anatid herpesvirus 1 challenge in ducks are currently being analyzed.

Fluorescence-based quantitative real-time PCR (qPCR) is commonly regarded as a straightforward, mature and ubiquitous means of molecular biology [37]. Probe-based qPCR technology is recommended as the reference technique for monitoring gene transfer biodistribution [38]. Therefore, in our previous study, we established a qPCR protocol based on the use of a TaqMan™ probe [39]. In contrast to previous studies, equivalent amounts of DNA (100 μg) were injected in the form of pcDNA3.1-gC, DNA-lipid complexes and chitosan-DNA nanoparticles and 200 ng of gDNA were used in each PCR reaction by preparation of the appropriate dilution ratios. Because we observed that there existed the inhibition due to the gDNA matrix, which affected quantification using qPCR (data not shown). This was consistent with other studies [40]. In the present study, 100 μg of plasmid was injected for each group. The same amount of each tissue was collected.

In summary, we successfully used chitosan and lipid formulations for DNA vaccination in ducklings. Compared with pcDNA3.1-gC alone, chitosan/DNA and DNA/lipid complexes improved the efficiency of plasmid distribution. The complexes were rapidly absorbed, and extensive and relatively long-term distribution at low concentrations was observed following DNA vaccination in ducklings.

## Conclusions

In this study, we succeeded in constructing a eukaryotic expression plasmid encoding the AnHV-1 gC gene. Endonuclease digestion and indirect immunofluorescence confirmed the identity of the construct and quantitative real-time PCR analysis demonstrated rapid and extensive distribution of the plasmid, which was subsequently maintained at a low level after one day. Thus, chitosan and lipid mediate efficient plasmid distribution and facilitate long-term maintenance of the plasmid.

## Methods

### Plasmid preparation

As shown in Figure 1a, pcDNA3.1-gC, which encoded the AnHV-1glycoprotein C, was generated by cloning gC gene into the *Eco*R I and *Xho* I sites of pcDNA3.1(+) (Invitrogen, USA) vector according to the manufacturer’s protocol. The purified gC gene was obtained from pMD18-gC [41] by restriction enzyme digestion (*Eco*R I and *Xho* I) (Takara, Japan). The pcDNA3.1-gC plasmid was transformed into *E. coli* JM109. By means of a fed-batch process for high cell density cultivation, *E. coli* harboring pcDNA3.1-gC was produced using a Biostat® Cplus fermenter (Sartorius, Germany). The fermentation broth was centrifuged at 5000 × *g* for 10 min in an Avanti J-26XP Centrifuge (Beckman Coulter, USA). Preparation and purification of plasmid DNA were based on alkaline lysis with SDS (maxipreparation) and precipitation with polyethylene glycol as described by Sambrook [42]. The purity of plasmid DNA was assessed by agarose gel electrophoresis and using the 260 nm and 280 nm absorbance ratio (A_260/280_). The purified DNA was stored at −20°C.

### Transient expression and indirect immunofluorescence (IIF) assay

The pcDNA3.1-gC was first transfected into COS-7 cells using Lipofectamine™ 2000 (Invitrogen, USA) according to the manufacturer’s instructions. Briefly, plasmid DNA (4 μg) was diluted with 250 μL serum-free DMEM media. Lipofectamine (10 μL) was diluted with the same media. The diluted plasmid DNA and Lipofectamine solutions were then mixed. The DNA-Lipofectamine solutions were added to six-well culture plates containing coverslips. After incubation at 37°C for 48 h, cells were fixed with 4% paraformaldehyde. After blocking for 1 h in phosphate-buffered saline (PBS) containing 10% BSA at 37°C, the coverslip was incubated with the anti-gC IgG antibodies (1:100) at 4°C overnight, and then treated with FITC-conjugated goat anti-rabbit IgG (Sino-American Biotechnology Co., China) (1:100) for 45 min at 37°C. The stained cells were examined with the Nikon 80i fluorescence microscope (Tokyo, Japan).

### Preparation of DNA-lipid complexes (designated lipoplex-gC)

DNA-lipid complexes were prepared as described by Rosada [43] and Torchilin [44]. The required amounts of all lipid stock solutions in chloroform (PC/CH/ODA 5:4:1 molar) were mixed and dried to a thin film using a rotary evaporator in vacuum for 1 h. The films were hydrated in sterile water above its phase transition temperature. The hydrated lipid solution was then sonicated and passed sequentially through two polycarbonate membranes (0.45 μm nominal diameter and 0.22 μm nominal diameter; 15 times per membrane). DNA (pcDNA3.1-gC) was mixed with the extruded liposomes at a lipid/DNA mass ratio of 1/50, frozen, and vacuum freeze-dried overnight. Controlled rehydration of the dry powders with PBS resulted in the formation of liposomes encapsulating the DNA.

### Preparation of chitosan-DNA nanoparticles (designated chitosan-gC polyplexes)

Chitosan (MW 55 kDa, deacetylation degree 90%, medical grade) was kindly provided by Golden-Shell Biochemical Co., Ltd (China). It was purified by reprecipitation from the filtered 1% acetic acid solution with ammonium hydroxide. The precipitate was washed with water and dried under vacuum. The purified chitosan was dissolved in 1% acetic acid with gentle heating and the pH of the solution was adjusted to 5.5–5.7 with sodium hydroxide. The solution was diluted to 0.03% chitosan (w/v) and 5 mM acetate. The pH of the chitosan stock solution was readjusted to pH5.5 and sterile filtered through a 0.22 μm filter. The solution was stored at 4°C. A chitosan solution (0.03% in 5 mmol/L sodium acetate buffer, pH 5.5) and a DNA solution (100 μg/mL in 10 mmol/L sodium sulfate solution) were preheated to 55°C separately. An equal volume of both solutions were quickly mixed together and vortexed for 15–30 s (amino group to phosphate group ratio (N/P ratio) of 3:1). The final volume of the mixture in each preparation was limited to a maximum of 500 μL in order to yield uniform nanoparticles. The homogeneity of chitosan-gC polyplex nanoparticles was analyzed by transmission electron microscopy (Hitachi H-600, Tokyo, Japan) as described by Jiang [45].

### Animal vaccination and sample collection

This study was conducted with 80 AnHV-1-free Peking ducks (28 days old) from an AnHV-1-free farm. The AnHV-1 status was confirmed by qualitative PCR as described by Song [46]. The ducks were fed with commercial duck pellets. Water troughs that were deep enough for the ducks to float and splash were placed in each room. Each room also had a partially enclosed dry retreat with wood shavings for the ducks to sit on. The ducklings were randomly divided into four equal groups in this study. Using 5 mL syringes, ducks in groups 1–3 were inoculated by the intramuscular route with pcDNA3.1-gC alone, lipoplex-gC, and chitosan-gC polyplex each containing equivalent amounts of plasmid DNA (100 μg). Ducks in group 4 were inoculated with pcDNA-3.1(+) as a control. At each of eight sampling times, two vaccinated ducks per group were chosen randomly for sampling. The liver, spleen, kidney, lung, trachea, esophagus, heart, brain, duodenum, rectum, cecum, injection site and bursa of Fabricius were collected at 1 h, 4 h, 8 h, 12 h, 1 d, 7 d, 4 weeks and 10 weeks post-inoculation. Each solid tissue sample was transferred to a labeled bag, snap-frozen in liquid nitrogen, and stored at −80°C. All animal work was conducted with the approval of the Sichuan Agricultural University Animal Ethics Committee.

### DNA extraction from tissues

Total DNA was isolated from each of the harvested tissues by phenol-chloroform extraction [47]. Tissue (50 mg) was minced and suspended in 500 μL proteinase digestion buffer (10 mmol/L Tris-HCl, 0.1 mol/L EDTA, 0.5% SDS, 20 μg/mL RNase A, pH 8.0) and homogenized using a tissue homogenizer (IKA, Germany). After incubation at 37°C for 1 h, homogenized tissue was digested with proteinase K (100 μg/mL) overnight at 55°C, extracted three times with equal volumes of buffered phenol and chloroform and precipitated with 2.5 volumes of ethanol. DNA was pelleted by centrifugation at 13,000 × *g* for 10 min, rinsed in 70% ethanol and air dried under an isolation hood before being resuspending in 50 μL TE buffer (pH 8.0). DNA concentrations were determined by UV absorption at 260 nm. The A_260/280_ absorbance ratio for each sample was confirmed to be ≥1.8. Each sample was analyzed by electrophoresis in a 0.8% agarose gel to ensure that the DNA migrated predominantly as a high molecular weight band and exhibited no detectable RNA contamination. The tissue DNA was stored at −20°C for further analysis.

### Quantitative real-time PCR assays

The levels of gC in negative and positive control DNA (from non-injected or pcDNA3.1-gC), and test sample DNA were quantified by a qPCR assay as described previously [39]. The primers used for PCR amplification were forward primer (FP), 5^′^-GAAGGACGGAATGGTGGAAG-3^′^, and reverse primer (RP), 5^′^-AGCGGGTAACGAGATCTAATATTGA-3^′^, which amplify a 78 bp fragment of the AnHV-1 gC gene (GenBank; Accession No.: EU076811). A 23-bp TaqMan probe (FAM-CCAATGCATCGATCATCCCGGAA-TAMRA) complementary to an internal region between the two primers was selected and synthesized with a FAM (6-carboxyfluorescein) reporter molecule at the 5^′^end and a TAMRA (tetra-methylcarboxyrhodamine) quencher molecule at the 3^′^end. The qPCR reactions were carried out using a Premix Ex Taq™ kit (Takara, Japan) with an iCycler iQ™ Multicolor Real-Time PCR Detection System (Bio-Rad, USA). All qPCR assays were performed using the same format. The amplification was performed in a 20 μL reaction mixture containing 2.0 μL DNA solution (200 ng of tissue DNA was used by preparing different dilution ratios), 10 μL 2 × Premix Ex Taq™, 0.5 μmol/L each primer and 0.25 μmol/L fluorogenic probe. The two-step PCR cycling conditions were as follows: initial denaturation and hot-start Taq DNA polymerase activation at 95°C for 5 min, 45 cycles of denaturation at 94°C for 5 s, primer annealing and extension at 53°C for 30 s with fluorescence acquisition during each annealing and extension stage. The tests were carried out by using the 0.2 mL PCR tubes (Axygen, USA). The reaction, data acquisition, and analysis were performed using iCycler iQ™ Optical detection system software (Version 3.1). A plasmid DNA sample was diluted to contain 1 × 10^1^–1 × 10^8^ copies per test tube and used as a standard series.

## Competing interests

The authors declare that they have no competing interests.

## Authors’ contributions

KFS, XL, JFJ carried out most of the experiments. KFS, XL wrote the manuscript. ACC and MSW critically revised the manuscript and designed the experiments. DKZ, RYJ, SC, YZ, XYC and XYW helped to perform the experiments and wrote the manuscript. All authors read and approved the final manuscript.
